# Sulforaphane Protects Rodent Retinas against Ischemia-Reperfusion Injury through the Activation of the Nrf2/HO-1 Antioxidant Pathway

**DOI:** 10.1371/journal.pone.0114186

**Published:** 2014-12-03

**Authors:** Hong Pan, Meihua He, Ruixing Liu, Nicholas C. Brecha, Albert Cheung Hoi Yu, Mingliang Pu

**Affiliations:** 1 Neuroscience Research Institute, Department of Neurobiology, School of Basic Medical Sciences, Peking University, Beijing, China; 2 Department of Anatomy/Embryology, School of Basic Medical Sciences, Peking University, Beijing, China; 3 Department of Physiology, Binzhou Medical College, Yantai, Shandong, China; 4 Key Laboratory on Machine Perception (Ministry of Education), Peking University, Beijing, China; 5 Key Laboratory for Visual Impairment and Restoration (Ministry of Education), Peking University, Beijing, China; 6 Department of Neurobiology, Department of Medicine, Jules Stein Eye Institute, CURE Digestive Diseases Research Center, David Geffen School of Medicine at Los Angeles, University of California Los Angeles, Los Angeles, California, United States of America; 7 Veterans Administration Greater Los Angeles Health System, Los Angeles, California, United States of America; Emory University, United States of America

## Abstract

Retinal ischemia-reperfusion (I/R) injury induces oxidative stress, leukocyte infiltration, and neuronal cell death. Sulforaphane (SF), which can be obtained in cruciferous vegetables such as broccoli, exerts protective effects in response to oxidative stress in various tissues. These effects can be initiated through nuclear factor E2-related factor 2 (Nrf2)-mediated induction of heme oxygenase-1 (HO-1). This investigation was designed to elucidate the neural protective mechanisms of SF in the retinal I/R rat model. Animals were intraperitoneally (i.p.) injected with SF (12.5 mg/kg) or vehicle (corn oil) once a day for 7 consecutive days. Then, retinal I/R was made by elevating the intraocular pressure (IOP) to 130 mmHg for 1 h. To determine if HO-1 was involved in the Nrf2 antioxidant pathway, rats were subjected to protoporphyrin IX zinc (II) (ZnPP, 30 mg/kg, i.p.) treatments at 24 h before retinal ischemia. The neuroprotective effects of SF were assessed by determining the morphology of the retina, counting the infiltrating inflammatory cells and the surviving retinal ganglion cells (RGCs) and amacrine cells, and measuring apoptosis in the retinal layers. The expression of Nrf2 and HO-1 was studied by immunofluorescence analysis and western blotting. I/R induced a marked increase of ROS generation, caused pronounced inflammation, increased the apoptosis of RGCs and amacrine cells and caused the thinning of the inner retinal layer (IRL), and these effects were diminished or abolished by SF pretreatment. Meanwhile, SF pretreatment significantly elevated the nuclear accumulation of Nrf2 and the level of HO-1 expression in the I/R retinas; however, ZnPP reversed the protective effects of SF on I/R retinas. Together, we offer direct evidence that SF had protective effects on I/R retinas, which could be attributed, at least in part, to the activation of the Nrf2/HO-1 antioxidant pathway.

## Introduction

Retinal ischemia-reperfusion (I/R) injury is important in many diseases, including diabetic retinopathy, acute glaucoma, retinal artery occlusions, and retinopathy of prematurity [1]–[3]. Retinal I/R interrupts blood flow to retinas and results in a deficiency of oxygen and other nutrients, whereas the subsequent reperfusion exacerbates the tissue damage because of the generation of reactive oxygen species (ROS) that lead to oxidative stress and inflammation [1], [4]. Oxidative stress plays a vital role in retinal neuronal injury [5]–[6]; therefore, the identification of a potential antioxidant therapy for I/R-induced damage has attracted intense interest.

The antioxidant response element or electrophile response element (ARE or EpRE)-mediated antioxidant enzymes and phase II detoxifying enzymes are responsible for suppressing oxidative damage and maintaining cellular redox homeostasis [7]–[9]. Transcription factors such as nuclear factor E2-related factor 2 (Nrf2), which plays a crucial role in ARE-regulated gene expression [10], bind to ARE and transactivate the downstream target genes. Nrf2 belongs to a member of the cap ‘n’ family and is primarily targeted for proteasomal degradation through its cytosolic binding protein kelch-like ECH-associated protein 1 (keap 1) [11]–[13]. Under conditions of oxidative stress, Nrf2 is released from keap1, translocates into the nucleus and binds to AREs within the promoters of genes that encode antioxidant enzymes, including heme oxygenase-1 (HO-1), to offset cellular oxidative stress [14]–[16]. As a stress inducible and redox-sensitive protein, HO-1 exerts potent indirect anti-oxidative functions by degrading heme to carbon monoxide (CO), iron, and biliverdin [17]–[18]. Moreover, these byproducts have significant roles in essential cellular metabolism and contribute to the suppression of oxidative stress [17], [19], [20]. The cells isolated from HO-1^−/−^ mice are more highly susceptible to oxidative injury in vitro compared with cells from wild type mice [21]. HO-1 over expression protects the retina from cellular damage caused by I/R injury [22], [23].

Our previous studies have shown that Lycium barbarum polysaccharides (LBPs), which are wolfberry extracts, have protective effects on the rodent retinas by activating the Nrf2/HO-1 antioxidant pathway, which counteracts I/R-induced damage [24]. However, LBP is not easily purified and is not very well-characterized; therefore, we focused on other well-characterized antioxidative compounds. As an Nrf2 activator, sulforaphane (SF) is one of the most abundant isothiocyanates in several of the cruciferous vegetables, particularly broccoli [25]. It is well documented that SF has cytoprotective effects that lead to increased expression of multiple antioxidant proteins [26]. Numerous studies have demonstrated that SF protects the kidneys, heart, brain and liver against ischemic injury through the activation of the Nrf2-antioxidant response element pathway [26]–[29]. Moreover, in vivo and in vitro studies have shown that SF protects retinal pigment epithelial (RPE) cells against photo-oxidative or light damage by inducing the expression of phase 2 proteins or AREs [30], [31]. Although numerous studies have demonstrated the protective effects of SF in various diseases, the effects of SF on retinal I/R injury have not yet been defined. The objective of this study was to clarify whether SF has protective effects on retina neuronal cells against retinal I/R injury and to identify the related mechanisms involved in this process.

## Materials and Methods

### Animals

Male Sprague-Dawley rats (eight-week-old, weight around 300–350 g) were used in the present study. They were housed at a temperature range from 20–23°C under 12-h light/12-h dark cycles. The rats had free access to food and water. All experiments were performed in accordance with the Peking University guide lines for animal research and the ARVO Statement for the Use of Animal in Ophthalmic and Vision Research. The experimental animal protocol used in our study was approved by the Peking University Animal Care and Use Committee (IACUC).

#### The animal model of retinal I/R

Retinal I/R was induced by increasing the intraocular pressure (IOP), as described in our recent report [24]. Briefly, the rats were anesthetized using a cocktail of ketamine (80 mg/kg) and xylazine 8 mg/kg). The anterior chamber of the left eye was cannulated with a 27-gauge infusion needle connected to a physiological saline reservoir. The intraocular pressure (IOP) was increased to 130 mmHg for 60 minutes by elevating the saline reservoir. The successful achievement of retinal ischemia was confirmed by the collapse of the central retinal artery, which was identified by the whitening of the iris during the elevation of IOP [22]. After 60 minutes of ischemia, the needle was withdrawn and the IOP was normalized to allow reperfusion for 24 h or 7 days. The other eye of the same rat was used as the control and was not subjected to ischemic injury.

### Experimental design

D,L-Sulforaphane (SF, 12.5 mg/kg, dissolved in corn oil) [32] (Sigma-Aldrich Corp., St. Louis, MO)), or corn oil (vehicle) was administered through an intraperitoneal (i.p.) injection once daily for 7 consecutive days. All drug treatments were performed at 10 am each day. Twenty-four hours after the final injection of SF or vehicle, retinal I/R was induced. Protoporphyrin IX zinc (II) (ZnPP, 30 mg/kg; Sigma-Aldrich Corp., St. Louis, MO), an HO-1 inhibitor, was administered via an i.p. injection 24 h prior to retinal ischemia followed by 1 h of retinal ischemia. ZnPP was prepared by dissolving the compound in 1 ml of 0.1 M NaOH. The pH was adjusted to 7.4 with 1 M HCl and diluting the solution to the final volume with 0.9% NaCl [33]. Lycium barbarum (wolfberries) was purchased from a local supermarket located in the Ning Xia Huizu Autonomous Region, People's Republic of China. The dried wolfberries were ground into small pieces, delipidated and deproteinated in alcohol. LBP was then extracted using 70°C water as previously described [24]. The extracts were freeze-dried into a powder in phosphate-buffer saline (PBS; 0.01 M; pH 7.4). The animals were orally fed LBP (1 mg/kg) by gavage once a day for 1 week followed by 1 h of retinal ischemia. The rats were randomly assigned to seven experimental groups: the non-ischemic control group (the needle was inserted into the anterior chamber without elevating the IOP, control); the vehicle group (the animals were injected intraperitoneally with corn oil followed by retinal ischemia; I/R); the SF-pretreated group (the animals were injected intraperitoneally with SF followed by retinal ischemia; SF+I/R); the SF+ZnPP +I/R group (SF and ZnPP were injected intraperitoneally, followed by retinal ischemia; SF+ZnPP+I/R); the SF-pretreated control group (SF-pretreated rats in the absence of retinal ischemia; SF control); and the LBP+I/R group (the animals were orally fed by gavage with LBP followed by retinal ischemia;, LBP+I/R). The animals were sacrificed by administrating an overdose of sodium pentobarbital at 24 h or 7 days after ischemia.

#### Detecting the ROS Generation

The generation of retinal ROS was assessed using dihydroethidium (DHE; Invitrogen Molecular Probes, Eugene, OR), as described in our recent publication [34]. Briefly, after enucleation, fresh retinas were harvested and immediately snap-frozen in liquid nitrogen for cryosectioning (Leica CM1950; Leica Microsystems Ltd, Wetzlar, Germany). The cryosections (10 µm) were washed with a warm PBS solution and then incubated with 5 µM DHE in PBS for 30 minutes at 37°C. DHE specifically reacts with superoxide anions and is converted to the red fluorescent compound ethidium. The sections were examined and imaged using an inverted fluorescent microscope that was equipped with a digital camera (Eclipex Ti-S; Nikon Instech Co., Tokyo, Japan) under identical exposure conditions, and the optical densities of the staining in the outer nuclear layer (ONL), the inner nuclear layer (INL), and the ganglion cell layer (GCL) were measured in randomly selected images. Five measurements were taken at 200µm intervals from each image starting from the optic disk.

#### Immunofluorescence staining

The cryosections were used for immunofluorescence. Briefly, the eyeballs were enucleated and immersed in 4% paraformaldehyde for 60 minutes. After fixation, the eye cups were dehydrated using a gradient sucrose solution, and embedded in OCT compound (Sakura Finetek USA, Inc., USA). Cryosections (10 µm) were obtained along the temporal-nasal axis through the optic nerve head. The tissue specimens were blocked and permeabilized simultaneously with 3% BSA (Sigma-Aldrich Corp., St. Louis, MO) in 0.3% Triton X-100 for 1 hour at room temperature, and then incubated with primary antibodies specific for choline acetyltransferase (ChAT; Millipore Corp, Billerica, MA), RNA-binding protein with multiple splicing (Rbpms, an RGC marker; ProSci, Poway, CA), HO-1 (Stressgen, Inc., San Diego, CA) and Nrf2 (Santa Cruz Biotech Inc. Dallas, TX) at 4°C overnight, followed by incubation with FITC-labeled secondary antibodies (Abcam Inc., Cambridge, MA) for 1 hour at room temperature. Cell nuclei were counterstained with 4′-6-diamidino-2-phenylindole (DAPI). The retinal sections were examined using an inverted fluorescence microscope. For quantification, three retinal sections from each eye were collected to increase data reliability of the data. The ChAT-positive amacrine cells [35] and Rbpms-positive ganglion cells [36], [37] in both GCL and INL, and cells with Nrf2 nuclear accumulation in the GCL in each section were counted under fluorescence microscopy. For Nrf2 quantification, images of Nrf2 staining (green) and DAPI (blue) of the same retinal area merged to identify the cells with nuclear Nrf2 accumulation. Using the software Image J, the optical densities of the HO-1 staining in the GCL were tested in randomly selected images, and five measurements were taken at 200µm intervals from each image started from the optic disc.

#### TUNEL assay

Retinal cell apoptosis was examined using a terminal deoxynucleotidyl transferase (TdT)-dUTP nick end labeling (TUNEL) IHC kit (Life Technologies, Grand Island, NY) according to the manufacturer's protocol. The eyeballs and frozen sections were prepared as described above. At room temperature, after incubation with 0.3% Triton X-100 for 20 minutes for permeabilization, the sections were incubated with TdT reaction buffer for 10 minutes. Then, the sections were incubated with TdT reaction cocktail for 60 minutes at 37°C, and washed twice with 3% BSA in PBS for 2 minutes each. Sections were visualized on an inverted fluorescent microscope, and the TUNEL-positive cells in both GCL and INL in each section were counted.

The analysis of the inner retinal layer thickness and inflammation Hematoxylin and eosin (H&E)-stained sections were collected to determine the thickness of the inner retinal layer (IRL) and to count the number of inflammatory cells. The IRL is confined by the internal limiting membrane (ILM) and the border between the ONL and outer plexiform layer [38]–[40]. To avoid sampling bias, we only selected cryosections containing the optic nerve stump, and three discontinuous retinal sections from each eye were examined. The thickness of the IRL was measured using Image J software. For counting inflammatory cells, H&E-stained sections were visualized under a light microscope, and the number of the infiltrating inflammatory cells in the vitreous side was quantified in three discontinuous retinal sections per animal. The counting of the infiltrating inflammatory cells, the surviving RGCs, amacrine cells, and of the TUNEL positive nuclei group was performed by a naïve observer who had no knowledge of the treatment group.

#### Western Blotting

Western blotting analyses was performed as described in our previous publication [24]. Briefly, within two minutes after enucleation, the retinas were quickly isolated and shock frozen at −80°C. Then, the retina was sonicated in RIPA buffer (Santa Cruz Biotech Inc. Dallas, TX). The protein concentrations were measured using a BCA protein assay. Then, the proteins were transferred to nitrocellulose membranes (Millipore Corp, Billerica, MA). After blocking with 5% BSA for 1 hour at room temperature, the membranes were incubated with primary antibodies at 4°C overnight, including goat polyclonal antibody against ChAT (Millipore Corp, Billerica, MA), rabbit polyclonal antibody against HO-1 (Stressgen Biotech Inc., Philadelphia, PA.), and rabbit polyclonal anti–β-actin (Sigma-Aldrich Corp). Next, the membranes were washed and incubated with horseradish peroxidase-conjugated an anti–goat secondary antibody and an anti–rabbit secondary antibody (PerkinElmer, Inc., Wellesley, MA) for 1 h at room temperature. The Amersham Biosciences ECL Western blotting detection reagent (GE Healthcare Life Science, Uppsala, Sweden) was used for signal detection according to the manufacturer's protocol. The optical density value (OD) of each band was measured using software Image J.

#### Statistical Analyses

The data are expressed as the means ± SEM. Analysis between multiple groups for time course studies was performed by two-way ANOVA, with time course (1 day and 7 days) as one factor and pretreatment (control, I/R and SF+I/R) as the other factor, followed by Bonferroni post hoc tests. One-way ANOVA followed by Bonferroni post hoc tests was used to compare single variable data. Differences were considered significant at P<0.05.

## Results

### SF pretreatment attenuated retinal I/R-induced ROS generation

**Figure 1:**
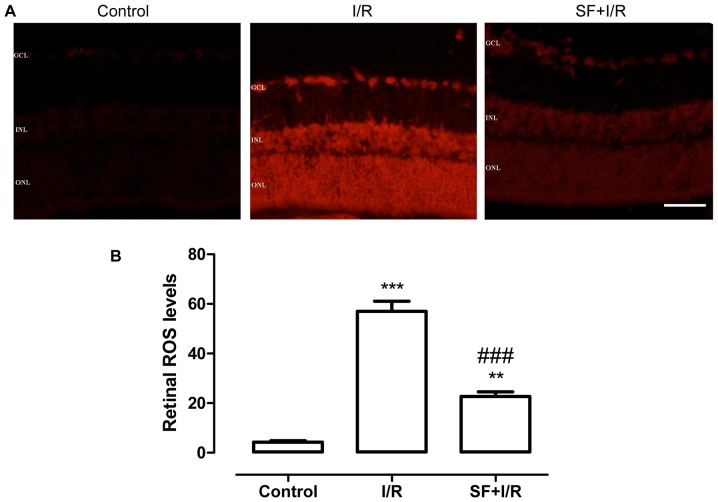
SF pretreatment attenuated generation in the retinal after I/R injury. ROS generation in fresh retinas was tested by DHE staining. (**A**) Representative micrographs of retinal sections stained with DHE (24 h after retinal ischemia). (**B**) Quantification analysis of ROS levels in the whole retina. The fluorescent intensities of DHE-labeled neurons were quantified using an image analysis software program (Image J; mean ± SEM, n = 5). Control: sham-operated animal, I/R: vehicle-treated animal with 1 h of ischemia, and SF+I/R: SF-pretreated rats with 1 h of ischemia. ** *p*<0.01, *** *p*<0.001 compared with control, ### *p*<0.001 compared with I/R, one-way ANOVA with Bonferroni post hoc test. Scale bar,50 µm. GCL, ganglion cell layer, INL, inner nuclear layer; ONL, outer nuclear layer.

The generation of excessive free radicals is well recognized as a critical primary event in retinal I/R injury [4]. ROS generation in fresh retinas was detected by DHE staining. As shown in Fig. 1 A, the non-ischemic control retinas exhibited a low level of detectable ROS. DHE fluorescence was significantly increased in the I/R retinas (p<0.001, one-way ANOVA, Bonferroni post hoc test), indicating an increase in the retinal superoxide levels in these animals. However, in the SF-pretreated animals, the level of DHE fluorescence in their retinas was significantly lower (p<0.001, one-way ANOVA, Bonferroni post hoc test;) (Fig. 1B).

### SF pretreatment inhibited inflammation after retinal I/R injury

**Figure 2:**
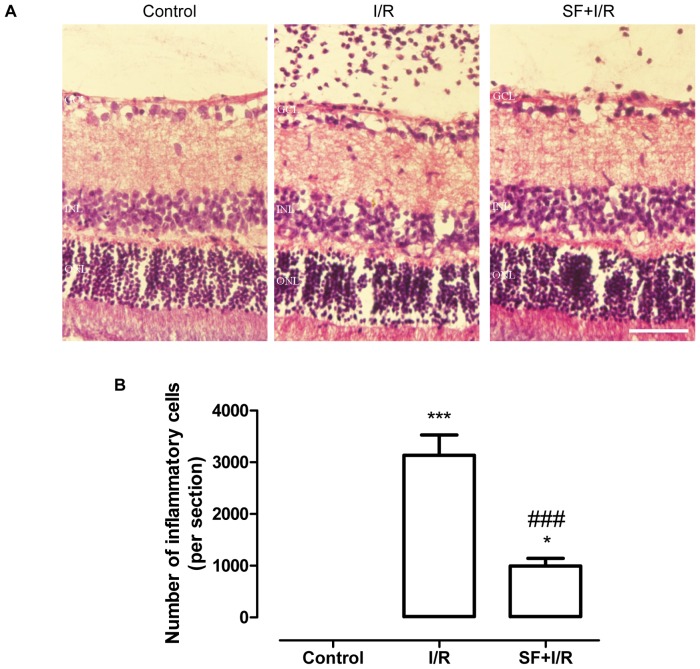
SF pretreatment inhibited inflammation after retinal I/R injury. The eyes were harvested and subjected to H&E staining for histological analysis at 24 h after I/R. (**A**) Representative micrographs of retinal sections with H&E staining (24 h after retinal ischemia). (**B**) The quantitative analysis of the infiltrating leukocytes in the retinas (mean ± SEM, n = 5). Control: sham-operated animals, I/R: vehicle-treated animal with 1 h of ischemia, and SF+I/R: SF-pretreated rats with 1 h of ischemia. * *p*<0.05, *** *p*<0.001 compared with control, ### *p*<0.001 compared with I/R, one-way ANOVA with Bonferroni post hoc test. The conventions are the same as in Figure 1.

Retinal inflammation is one of the well-described consequences of I/R injury. As shown in Fig. 2A, the histological features of the non-ischemic control retinas showed no significant histological changes. However, ischemia for 1 h and reperfusion for 24 h resulted in a significantly greater number of leukocytes infiltrating into the vitreous (p<0.001, one-way ANOVA, Bonferroni post hoc test). In contrast, the leukocyte infiltration was significantly decreased in the SF-treated I/R retinas (p<0.001, one-way ANOVA, Bonferroni post hoc test;) (Fig. 2B), indicating that SF has an anti-inflammatory role in the posterior chamber.

### SF pretreatment inhibited I/R- induced apoptosis of retinal cells

**Figure 3:**
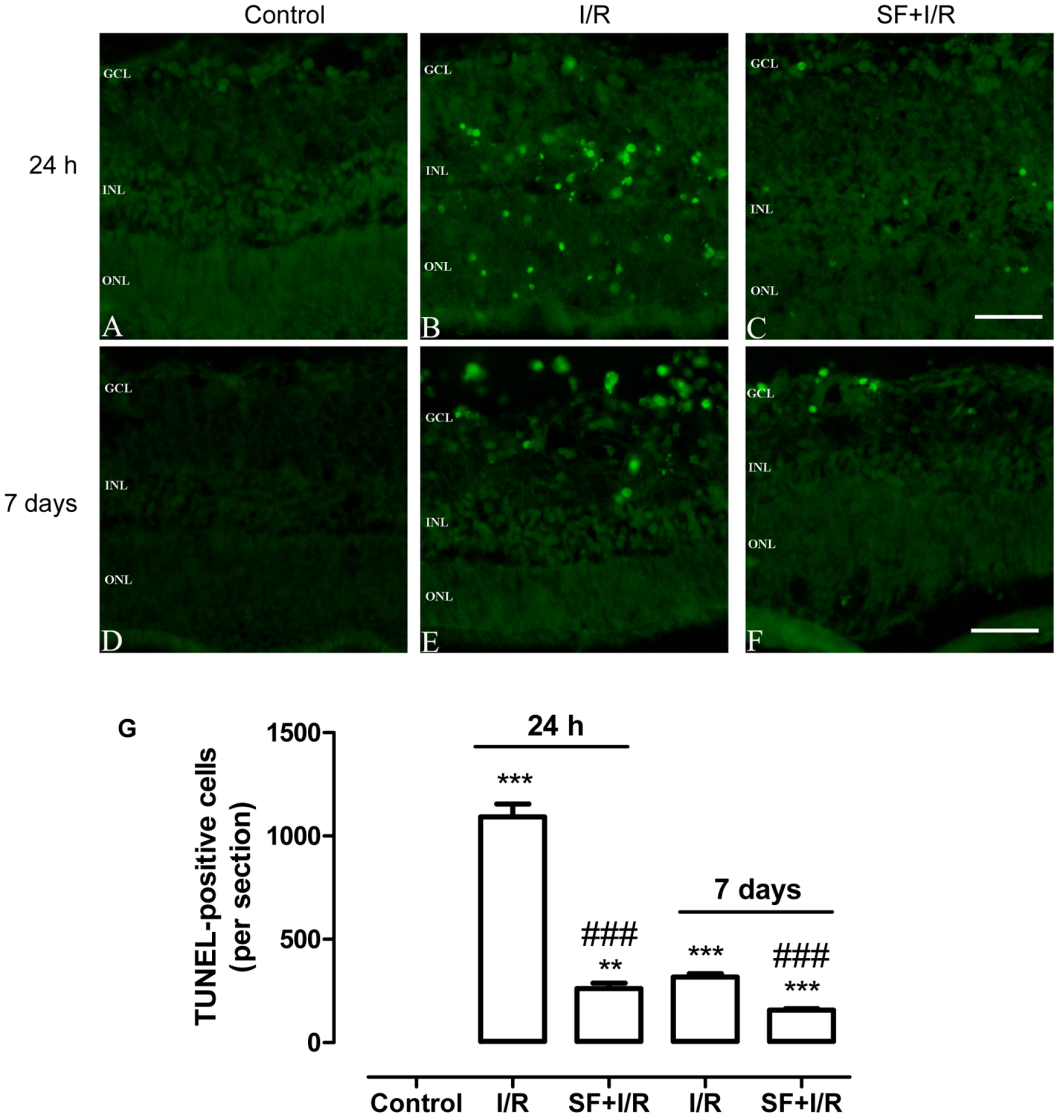
SF pretreatment inhibited I/R inducedretina cell apoptosis. The apoptotic cells in the retina were stained using a TUNEL -kit as described in the Methods section. (A-C, D-F) Representative micrographs of retinal sections obtained 24 h (A-C) or 7 days (D-F) after ischemia and stained for TUNEL. (G) Quantitative analysis of the TUNEL-positive cells in the retina (mean ± SEM, n = 5). Control: sham-operated animal, I/R: vehicle-treated animal with 1 h of ischemia, and SF+I/R: SF-pretreated animal with 1 h of ischemia. ** p<0.01, *** p<0.001 compared with control, ### p<0.001 compared with I/R within the same time point, two-way ANOVA with Bonferroni post hoc test. The conventions are the same as in Figure 1.

Apoptosis contributes to retinal neuron loss in an I/R injury animal model [41]. As shown in Figure 3, ischemia for 1 h and reperfusion for 24 h (Fig. 3 A-B) or 7 days (Fig. 3 D-E) resulted in significant increases in TUNEL-positive cells in the retina (24 h: p<0.001, two-way ANOVA, Bonferroni post hoc test; 7 days: p<0.001, two-way ANOVA, Bonferroni post hoc test), primarily in the INL and GCL, indicating that I/R leads to cell apoptosis in the retina. Fig. 3G depicts significantly fewer TUNEL-positive cells (24 h: p<0.001, two-way ANOVA, Bonferroni post hoc test; 7 days: p<0.001, two-way ANOVA, Bonferroni post hoc test) were observed in the INL and GCL of SF-pretreated retinas compared with the vehicle-treated retinas at both 24 h (Fig. 3C) and 7 days (Fig. 3F) after I/R, suggesting that pretreatment with SF for 1 week significantly protected retinal cells against I/R-induced damage and that this protective effect persisted for at least 7 days.

### SF pretreatment protected RGCs in retinal I/R injury

**Figure 4:**
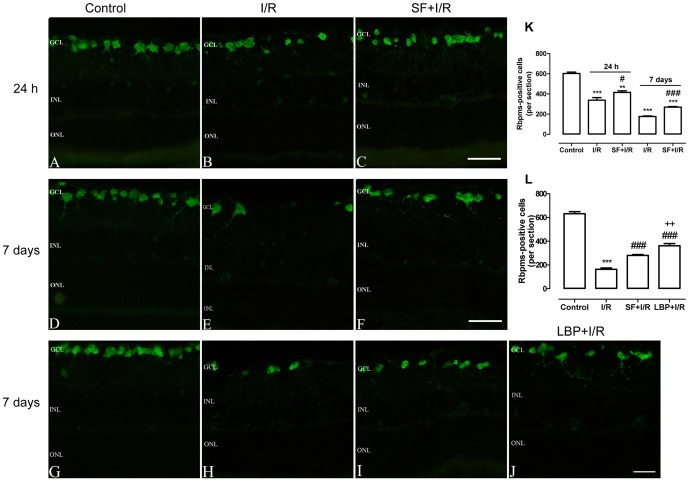
SF pretreatment protected RGCs from I/R induced damage. RGCs were stained with a specific marker, Rbpms. (A-J) Representative micrographs of retinal sections obtained 24 h (A-C) or 7 days (D-J) after ischemia and stained with anti-Rbpms. (K) Quantitative analysis of Rbpms-positive cells from experiments in (A-F) (mean ± SEM, two-way ANOVA with Bonferroni post hoc test, n = 5). (L) Quantitative analysis of Rbpms-positive cells from experiments in (G-J). (mean ± SEM, one-way ANOVA with Bonferroni post hoc test, n = 5). Control: sham-operated animal, I/R: vehicle-treated animal with 1 h of ischemia, SF+I/R: SF-pretreated animal with 1 h of ischemia, and LBP+I/R: LBP-pretreated animal with 1 h of ischemia. ** p<0.01, *** p<0.001 compared with control, # p<0.05, ### p<0.001 compared with I/R, ++ p<0.01 compared with SF+I/R within the same time point. The conventions are as for Figure 1.

To demonstrate whether SF pretreatment has protective effects on the RGCs in the I/R retina, Rbpms, a specific RGCs marker [36]–[37], was used to quantify the number of RGCs. As shown in Fig. 4A-B, a decline in the number of RGCs 24 h after I/R injury was observed, and the reduction continued 7 days after the injury (Fig. 4D-E). The progressive decrease in the number of RGCs was consistent in all examined retinas; therefore, we quantified the I/R-induced reduction in the number of RGCs. As shown in Fig. 4K, ischemia for 1 h and reperfusion for 24 h induced a 44% decline in the number of RGCs, and the reduction reached 71% at 7 days after the ischemia. However, the SF-pretreated animals showed clear resistance to the I/R injury, with a 31% and 55% decline at 24 h or 7 days after the injury, respectively (Fig. 4C, F). In addition, as exhibited in Fig. 4 G and H, I/R resulted in reduction in the number of RGCs, both SF and LBP pretreatments protected RGCs from I/R injury (Fig. 4I, J). However, in comparison with SF, the LBP-treated retinas showed a significantly higher number of surviving RGCs (SF, 304±15 vs. LBP, 374±37; p<0.01, one-way ANOVA with Bonferroni post hoc test,) (Fig. 4L).

### SF pretreatment protected retinal cholinergic amacrine cells from I/R damage

**Figure 5:**
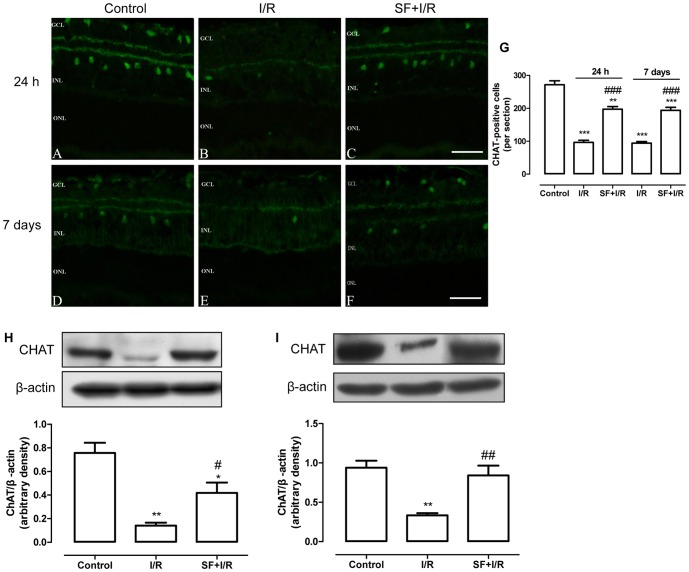
SF pretreatment protected ChAT positive amacrine cells in retinal I/R injury. Retinal amacrine cells were stained with an anti-ChAT antibody. (A-C, D-F) Representative micrographs of retinal sections obtained at 24 h (A-C) or 7 days (D-F) after ischemia and stained with an anti-ChAT antibody. (G) The quantitative analysis of ChAT-positive cells in the GCL and the INL. (H, I) Representative immunoblot of the ChAT levels in the whole retina (upper panel) at 24 h (H) or 7 days (I) after ischemia, and the densitometric analysis of ChAT expression relative to the loading control (lower panel) (mean ± SEM, n = 5). Control: sham-operated animal, I/R: vehicle-treated animal with 1 h of ischemia, and SF+I/R: SF-pretreated animal with 1 h of ischemia. * p<0.05, ** p<0.01, *** p<0.001 compared with control, # p<0.05, ## p<0.01, ### p<0.001 compared with I/R within the same time point, two-way ANOVA with Bonferroni post hoc test. The conventions are as for Figure 1.

The choline acetyltransferase (ChAT) antibody was used as a marker to label the cholinergic neurons in the retinas. As shown in Figure 5, in the non-ischemic control retinas, the ChAT-immunoreactive amacrine cells (ChAT-ACs) are clearly exist as two pairs of cells with mirror symmetry (Fig. 5A, D). The ChAT-a type cell has a cell body in the amacrine cell layer and its dendrites are monostratified in the sublamina a. In contrast, the ChAT-b type cell has a cell body displaced into the GCL and its dendrites stratified in the sublamina b. At 24 h after I/R, the number of ChAT-ACs in the two cellular layers was substantially reduced compared with the non-ischemic control retinas (Fig. 5B). Compared with the vehicle-treated I/R retinas, **t**he number of ChAT-ACs was increased in the SF-pretreated I/R retinas (Fig. 5C, G). Unlike the rate of progressive decline in the number of RGCs described above, the same rate of reduction (65%) in ChAT-immunoreactive cells was observed in the retinas at 7 days after ischemia, and the number of ChAT-ACs increased in the SF-pretreated I/R retinas (Fig 5F,G). There was a 65% reduction in the ChAT-immunoreactive cells in the retinas at 24 h and 7 days after ischemia (Fig 5G). These results were confirmed by immunoblotting. As exhibited in Fig. 5H (24 h) and 5I (7 days), the levels of ChAT protein in the in the vehicle-treated I/R retinas were significantly less than the levels in the non-ischemic retinas, and SF pretreatment significantly up-regulated the ChAT levels in the retinas after I/R (24 h: p<0.05, 7 days: P<0.01, two-way ANOVA with Bonferroni post hoc text).

### SF pretreatment protected against the I/R-induced decrease of IRL thickness

**Figure 6:**
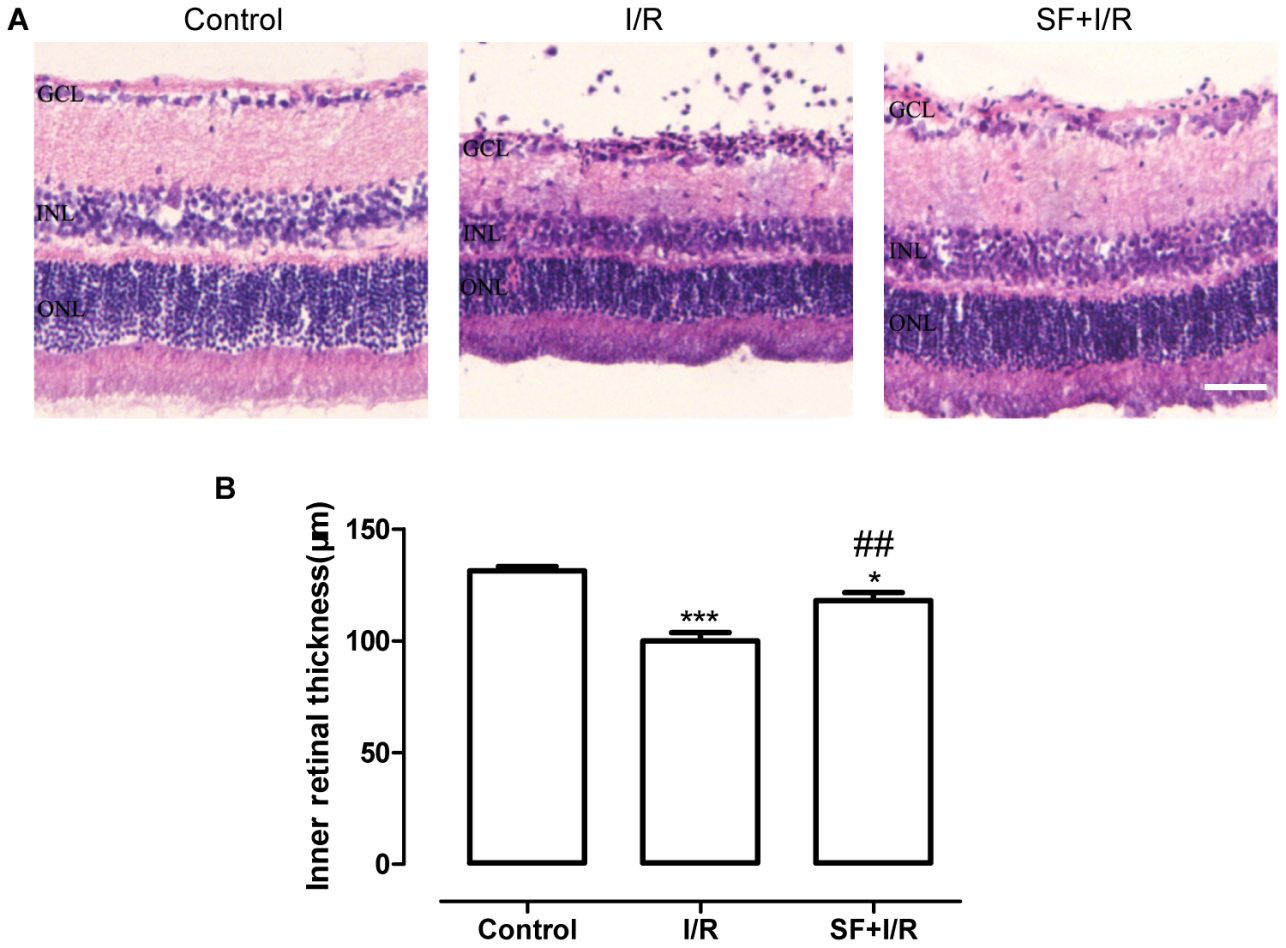
SF pretreatment inhibited the I/R induced decrease in the IRL thickness. H&E staining was performed to measure the IRL thickness at 7 days after ischemia. (**A**) Typical images of retinal sections from the middle area (7 days after retinal ischemia). (**B**) Quantitative analysis of the IRL thickness. (mean ± SEM, n = 5). Control: the non-ischemic rats, I/R: vehicle-treated rats with 1 h of ischemia, and SF+I/R: SF-pretreated rats with 1 h of ischemia. ** *p*<0.01, *** *p*<0.001 compared to control, ## *p*<0.01 compared to I/R, one-way ANOVA with Bonfferoni post hoc test. The conventions are the same as in Figure 1.

The I/R-induced damage to the retina was further assessed by measuring the IRL thickness. At 7 days after retinal ischemia (Fig. 6A), the IRL thickness in vehicle-treated I/R rats was decreased compared with the thickness observed in the non-ischemic control animals. However, after SF pretreatment, the IRL thickness in the I/R rats was increased compared with the vehicle-treated I/R rats. As shown in Fig. 6B, quantitative analyses reveal that SF significantly increased the IRL thickness in animals after I/R induced damage (P<0.01, one-way ANOVA with Bonferroni post hoc test).

### SF pretreatment enhanced the nuclear translocation of Nrf2 in GCL cells of I/R retinas

**Figure 7:**
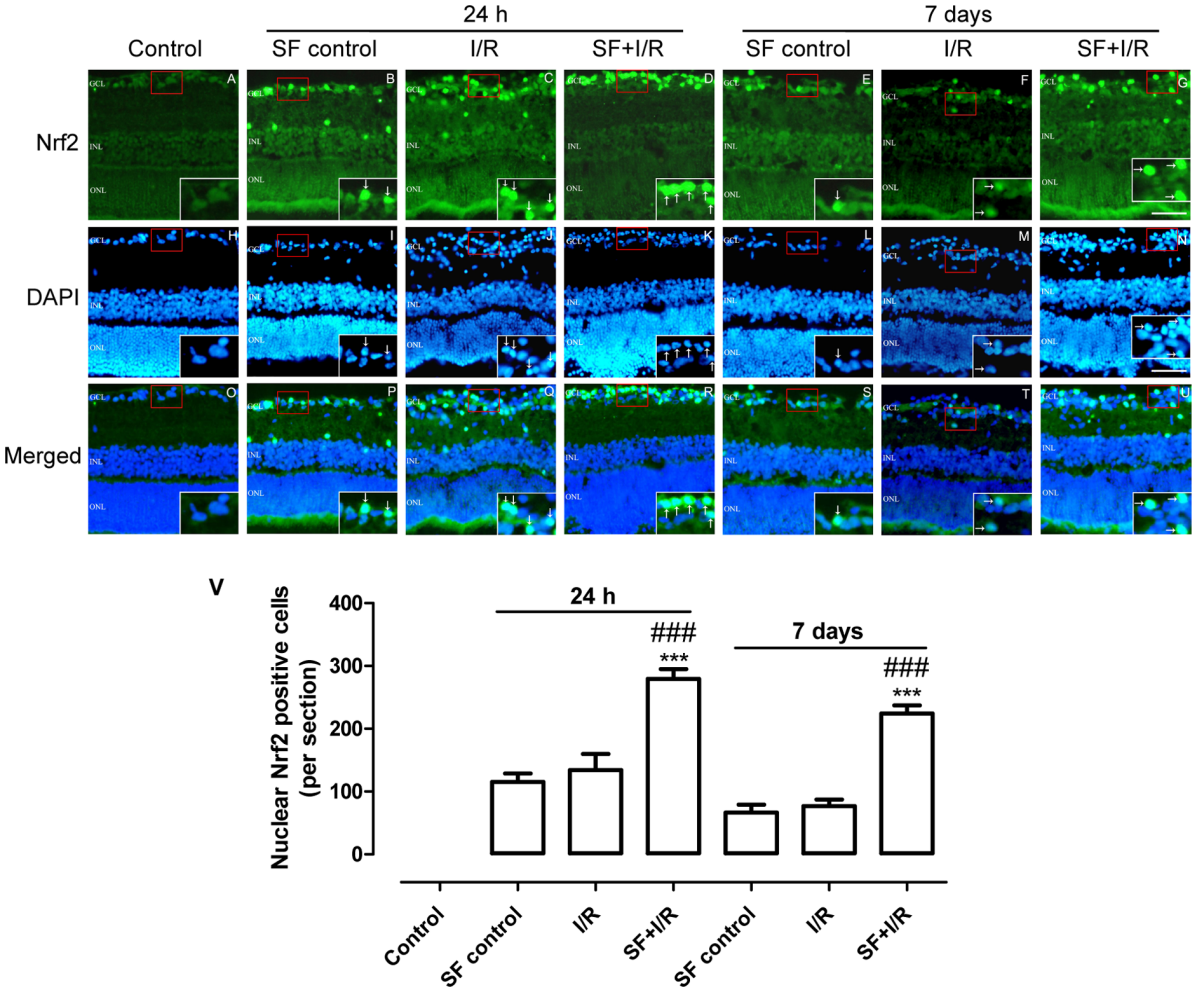
SF pretreatment enhanced the nuclear translocation of Nrf2 in GCL cells of I/R retinas. The Nrf2 localization and expression were of Nrf2 was determined by immunofluorescent staining using a specific anti-Nrf2 antibody. (A-D, E-G) Representative micrographs of retinal sections obtained 24 h (A-D) or 7 days (E-G) after ischemia and stained with anti-Nrf2. (H-N) Micrographs of DAPI counterstained nuclei obtained 24 h (H-K) or 7 days (L-N) after ischemia. (O-U) Micrographs of merged images obtained 24 h (O-R) or 7 days (S-U) after ischemia. Insets show magnified view of Nrf2-immunoreactive and DAPI double-stained somas of the retinal section marked with the red dotted line. White arrows point to double-labeled somas. (V) The quantitative analysis of nuclear Nrf2-positive cells in the GCL (mean ± SEM, n = 5). Control: sham-operated animal, I/R: vehicle-treated animal with 1 h of ischemia, and SF+I/R: SF-pretreated animal with 1 h of ischemia. ** p<0.01, *** p<0.001 compared with control, ### p<0.001 compared with I/R within the same time point, two-way ANOVA with Bonferroni post hoc test. The conventions are the same as in Figure 1.

Under oxidative stress, Nrf2 translocates into the nucleus and binds to ARE regions in the promoters of the genes that encode antioxidant enzymes to attenuate cellular oxidative stress [16]. Thus, we examined whether the improved survival rate of RGCs after SF pretreatment of the ischemic retinas was caused by Nrf2 up-regulation. As shown in Figure 7, in the non-ischemic control group, Nrf2 was detected diffusely in the cytoplasm and nuclei of the retinal cells (Fig 7A, H, O). Insets show magnified views of Nrf2-immunoreactive cell bodies of the retinal section marked with the red dotted line. In the SF control group and vehicle-treated I/R group (24 h after retinal ischemia), retinal neurons in the GCL had an increase in Nrf2 activation as indicated by the increased immunointensity of nuclear Nrf2 (Fig. 7B, I, P; Fig. 7C, J, Q). Pretreatment with SF further elevated Nrf2 translocation into the nucleus of the GCL cells (Fig. 7D, K, R). At 7 days after retinal ischemia injury, Nrf2 nuclear translocation was greatly diminished in the SF control (Fig. 7E, L, S) and I/R group (Fig. 7F, M, T) as indicated by the very few neurons exhibiting Nrf2 nuclear accumulation; however, in the SF-pretreated I/R retinas, cells with Nrf2 nuclear accumulation were clearly observed in the GCL (Fig. 7G, N, U). Quantification of the number of cells with nuclear accumulation of Nrf2 in the GCL demonstrated (Fig. 7V) that SF pretreatment significantly increased nuclear Nrf2 accumulation in I/R retinas at 24 h and 7 days after the I/R insult, (24 h: P<0.001; 7 days: P<0.001, two-way ANOVA with Bonferroni post hoc test).

### SF up-regulated the expression of HO-1 in I/R retinas

**Figure 8:**
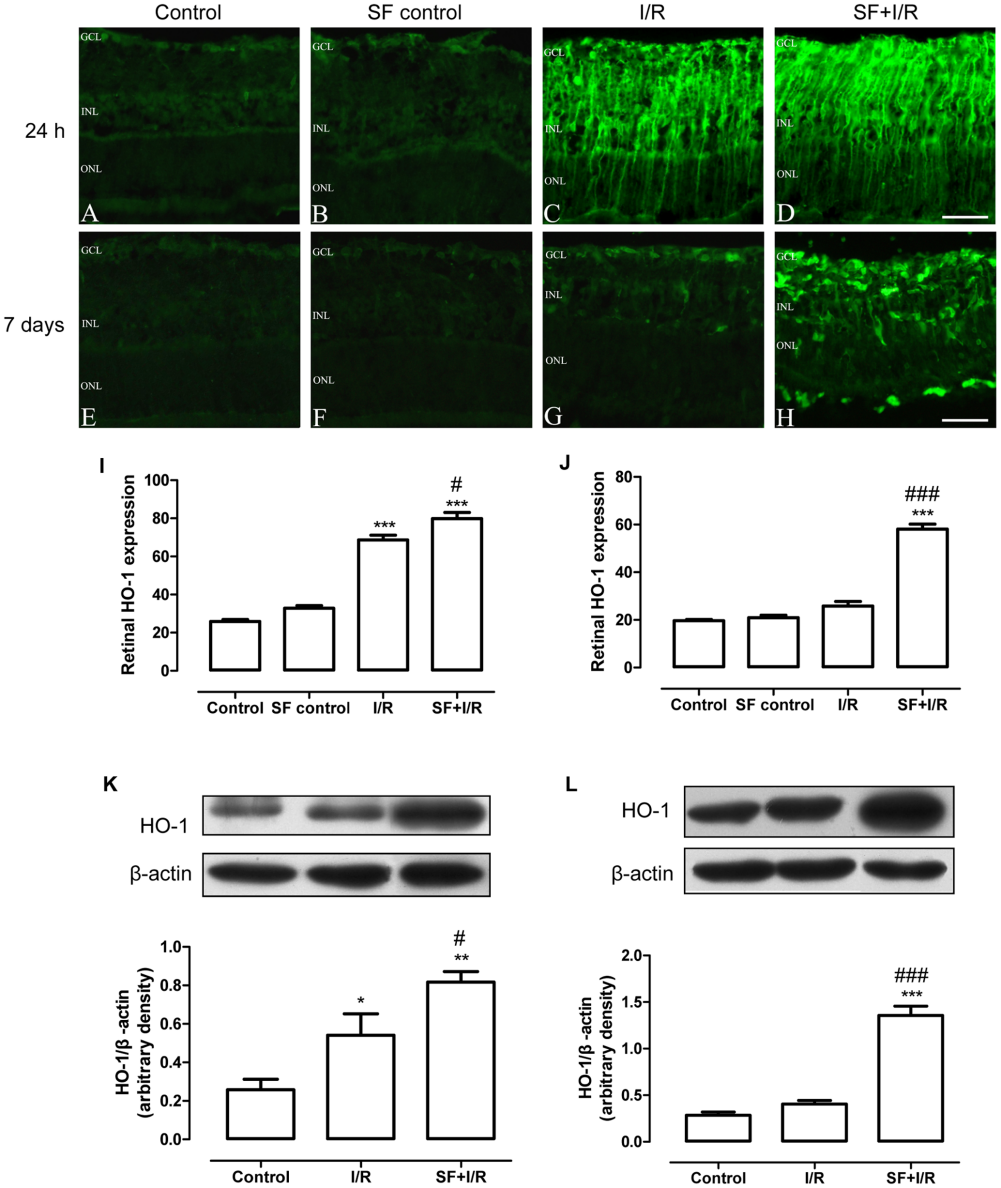
SF pretreatment elevated the HO-1 expression in I/R retinas. HO-1 expression in the retina was determined by immunofluorescent staining. (A-D, E-H) Representative micrographs of retinal sections obtained at 24 h (A-D) or 7 days (E-H) after ischemia and stained using an anti-HO-1 antibody. (I, J) Quantification of HO-1 immunofluorescent intensity (arbitrary units) at 24 h (I) or 7 days (J) after I/R injury. (K, L) Representative immunoblot showing the HO-1 protein levels in the whole retina (upper panel) at 24 h (K) or 7 days (L) after ischemia and densitometric analysis of HO-1 expression relative to the loading control (lower panel) (mean ± SEM, n = 5). Control: sham-operated animal, I/R: vehicle-treated animal with 1 h of ischemia, and SF+I/R: SF-pretreated animal with 1 h of ischemia. * p<0.05, ** p<0.01, *** p<0.001 compared with control, # p<0.05, ### p<0.001 compared with I/R within the same time point, one-way ANOVA with Bonferroni post hoc test. The conventions are the same as in Figure 1.

Nrf2 is a transcription factor that regulates the expression of HO-1 [13]. SF pretreatment induced cells to accumulate Nrf2 in the nucleus; therefore, the expression of one of the downstream target genes of Nrf2, HO-1, was then investigated by immunofluorescent staining and Western blot analysis. As shown in Fig. 8, the non-ischemic and SF controls had relatively low HO-1 immunoreactivity throughout the entire retina (24 h: Fig. 8A-B, 7 days: Fig. 8E-F). At 24 h after retinal ischemia, I/R induced stronger HO-1 immunoreactivity throughout the entire retina (Fig. 8C). Furthermore, pretreatment with SF enhanced HO-1immunoreactivity in the retinas after I/R (Fig. 8D). Quantification of immunofluorescent intensity (arbitrary units) showed a significant increase in HO-1 immunoreactivity in SF-pretreated retinal sections (SF+I/R) compared with I/R injury (I/R) and controls receiving vehicle (control) or SF pretreatment without I/R injury (SF-control; p<0.05, one-way ANOVA with Bonferroni post hoc test;) (Figure 8I). Of note, compared with control or SF-control, I/R induced significantly elevated HO-1 expression. Whereas, at 7 days after the I/R insult, HO-1 immunofluorescence intensity in the vehicle-treated I/R retina had almost diminished or returned to basal levels (Fig. 8G). Nevertheless, in the SF-pretreated I/R retina, HO-1 immunoreactivity remained strong throughout the entire retina (Fig 8H). This observation was confirmed by subsequent quantitative analysis (P<0.001, one-way ANOVA with Bonferroni post hoc test; Fig. 8J). Because there was no significant difference between the vehicle control and SF-pretreated control, we selected the vehicle control in immunoblotting experiments. The western blotting study revealed that, at 24 h after the I/R injury, the basal level of HO-1 in the non-ischemic retina was rather low. I/R induced a significant increase of HO-1 expression in the retina. SF pretreatment significantly enhanced the I/R induced increase in HO-1 expression (P<0.05, one-way ANOVA with Bonferroni post hoc test; Fig. 8K). At 7 days after the I/R injury, HO-1 expression approached basal levels. However, SF pretreatment significantly increased HO-1 expression (P<0.001, one-way ANOVA with Bonferroni post hoc test; Fig. 8L).

### ZnPP administration reversed the protective effects of SF on the I/R retinas

**Figure 9:**
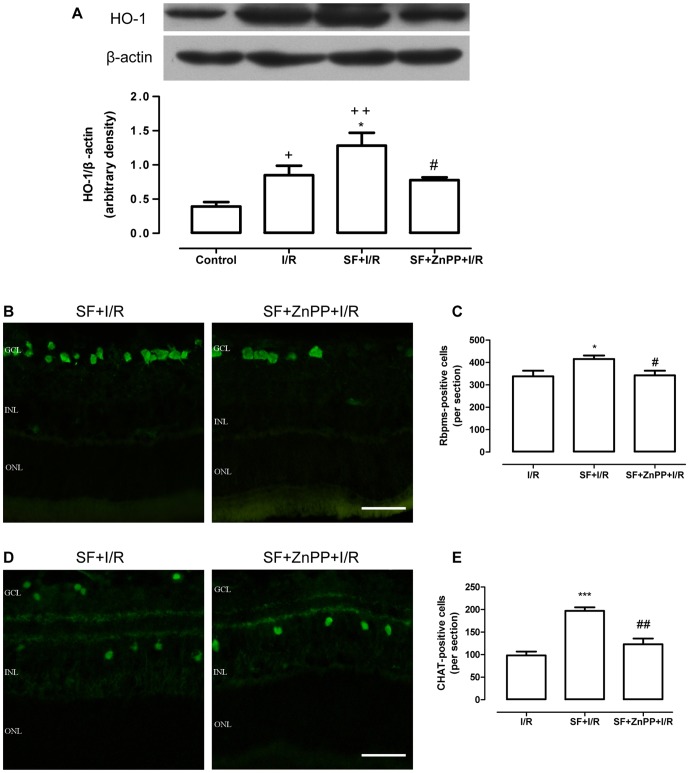
ZnPP administration reversed the protective effects of SF on the I/R retinas. The involvement of HO-1 in the protective effects of SF on the retina after I/R was determined using a specific HO-1 inhibitor, ZnPP. (A) Representative immunoblot of the HO-1 protein levels in the whole retina (upper panel) at 24 h after ischemia and densitometric analysis of HO-1 expression relative to the loading control (lower panel). (B) Micrographs of retinal sections obtained 24 h after ischemia and RGCs stained with Rbpms. (C) Quantitative analysis of the Rbpms-positive cells in the GCL (mean ± SEM, n = 5). (D) Micrographs of retinal sections obtained 24 h after ischemia and stained with an amacrine cell-specific marker, anti-ChAT. (E) Quantitative analysis of ChAT-positive cells in GCL and the INL (mean ± SEM, n = 5). Control: sham-operated animal, I/R: vehicle-treated animal with 1 h of ischemia, SF+I/R: SF-pretreated animal with 1 h of ischemia, and SF+ZnPP+I/R: SF-pretreated animal with ZnPP injection 24 h before 1 h of ischemia. + p<0.05, ++ p<0.01 compared with control, * p<0.05, *** p<0.001 compared with I/R, # p<0.05, ## p<0.01 compared with SF+I/R, one-way ANOVA with Bonferroni post hoc test. The conventions are the same as in Figure 1.

To investigate whether HO-1 protein expression was involved in the protective effects of SF on the RGCs and ChAT-ACs in the I/R retinas, a specific HO-1 inhibitor, ZnPP, was used in this study. As shown in Fig. 9A, ZnPP pretreatment significantly inhibited SF-induced increase in HO-1 expression in the I/R retinas (P<0.01, one-way ANOVA with Bonferroni post hoc test). Consequently, ZnPP pretreatment significantly reduced the number of Rbpms-expressing cells (P<0.05, one-way ANOVA with Bonferroni post hoc test; Fig. 9B-C) and ChAT-ACs (P<0.01, one-way ANOVA with Bonferroni post hoc test; Fig. 9D-E) in the SF+ZnPP+I/R group to the level observed in the vehicle-treated I/R retina. Together, these results provide evidence that the protective effects of SF on I/R retinas are at least partially mediated by HO-1.

## Discussion

This study shows that SF served as a neuroprotective agent against oxidative stress in I/R retinas. Specifically, I/R induced a marked increase in ROS generation, caused more pronounced inflammation, increased the apoptosis of RGCs and amacrine cells and reduced the thickness of the IRL. SF pretreatment not only alleviated or retarded the I/R induced retinal damage but also simultaneously enhanced Nrf2 activation and increased HO-1 protein expression in the I/R retinas. Moreover, SF-induced protective effects on I/R retinas were significantly reversed by the inhibition of HO-1 activity. These results suggest that SF is a potent antioxidant for I/R retinas, and the protective effects of SF were mediated, at least in part, by the activation of the Nrf2/HO-1 antioxidant pathway. To the best of our knowledge, this finding is the first direct evidence for a cytoprotective role of SF in the I/R retinas.

The retinal I/R model, which mimics clinical conditions such as acute-angle closure glaucoma and retinal artery occlusion, is widely used to study retinal neuronal cell damage after ischemic insult [22]. In this model, evidence of increased oxidative damage was observed [42]–[44], and the characteristic change involves the cell death of the RGCs and the inner retinal neurons, with the cell death levels peaking 7 days after the injury [45], [46]. The I/R injury induces cytotoxic responses including oxidative stress, pro-inflammatory responses, reduction of the IRL thickness, and neuronal cell death [38], [47], [48], which were all observed in our study. This model allowed us to investigate the role of SF specifically during oxidative stress/ischemia-mediated retinal injury.

SF is an isothiocyanate found in cruciferous vegetables such as broccoli and Brussels sprouts [49]. Numerous studies have shown that SF has anti-carcinogenic, anti-inflammatory, anti-apoptotic, and anti-oxidative functions in many types of tissues, including the liver, kidneys, heart and brain [26], [28], [49]–[53], and these functions are exerted via the activation and induction of phase 2 antioxidants and enzymes [28], [29], [54]. The protective effects of SF against ocular diseases have also been demonstrated. SF pretreatment protected the RPE and photoreceptor cells against light damage and delayed photoreceptor degeneration in tub/tub mice through Nrf2 up-regulation [31], [55]. Nevertheless, no study has shown the effect of SF on RGCs and retinal INL cells in the retinal I/R model. Numerous studies, including this one have demonstrated that retinal I/R causes the production of high ROS levels [1], [56], and the ROS attack nearby cells and cause tissue damage, leading to the death of the RGCs and the neurons in the inner retinal layer [46], [56]. Similar to the results found in other tissues, the present study demonstrated that SF pretreatment significantly inhibited ROS generation and alleviated the loss of RGCs and amacrine cells in the I/R retina, suggesting that SF has anti-oxidative and anti-apoptotic roles in retinas exposed to I/R. Furthermore, we observed that RGCs and ChAT-ACs responded differently to ischemic insult. As shown in Fig. 4 and 5, 24 h after I/R, the average number of RGCs was reduced by 44%, while the number of ChAT-ACs was reduced by 65%. Seven days after the insult, the RGC losscontinued; however, the number of ChAT-ACs ceased to decline. These results suggest that the death of RGCs and of ChAT-ACs is attributed to different mechanisms.

In addition to ROS generation, the consequent inflammation also induces tissue damage [4]. A recent study has shown that the blood-retinal-barrier (BRB) was severely damaged in I/R retinas [38], and the disruption of the BRB increases retinal vascular permeability, resulting in retinal edema and cell death [57], [58]. In the present study, we demonstrated that the I/R retinas exhibited a significant accentuation of leukocyte infiltration in the retina and the vitreous material, and SF pretreatment reversed this effect, suggesting that SF plays an anti-inflammatory role in the retina, possibly by maintaining the integrity of the BRB.

As an activator of Nrf2 and a potent inducer of phase II detoxification enzymes, SF exerts its protective effects via the activation of phase II antioxidants and detoxifying enzymes [27]–[29], [49], [54]. Among these enzymes, HO-1 has been reported to have the largest number of AREs present in its promoter, making it a highly effective therapeutic target for protection against neurodegenerative diseases [59]. Accumulating evidence suggests that HO-1 exerts potent endogenous anti-oxidative, anti-inflammatory and anti-apoptotic properties [17], [23]. HO-1 over expression by pharmacologic induction protects the retina from subsequent cellular damage caused by I/R injury [22]. Extensive studies have demonstrated that SF protects the kidneys, brain and liver against ischemic injury through increasing the expression of Nrf2 and HO-1 [27]–[29]. Consistent with these results, the present study also validated that SF pretreatment significantly increased the nuclear accumulation of Nrf2 and the expression of HO-1 in the I/R retinas. The persistence of Nrf2 and HO-1 expression deserves a brief comment. We observed that nuclear Nrf2 expression increasingly accumulated, and endogenous HO-1 was up-regulated in the retinas after I/R injury. However, this adaptive activation was temporary and diminished 7 days after the I/R injury. However, pretreatment with SF prolonged this effect for at least 7 days after the I/R injury. This result suggests that the increase Nrf2 and HO-1 expression induced by I/R injury was triggered by compensatory mechanisms against ROS in the animal. However, the protective effect induced by SF was initiatively activated to scavenge the harmful free radicals and enhance the antioxidant capacity of the cell. Concurrent with the enhanced expression of Nrf2/HO-1 that was induced by SF, the infiltration of inflammatory cells, and the apoptosis of RGCs and amacrine cells were also markedly inhibited by the pretreatment of SF. These results provide evidence that neuroprotective effects of SF on the I/R retinas are mediated through the activation of the Nrf2/HO-1 antioxidant pathway. Furthermore, as shown in Fig. 9, we demonstrated that ZnPP, an HO-1 inhibitor, pretreatment significantly inhibited HO-1 expression in the SF-pretreated I/R retinas and reversed the SF-induced inhibition of the apoptosis of RGCs and ChAT-ACs after I/R., These observations suggest that the Nrf2/HO-1 antioxidant pathway is involved in the neuroprotective effects of SF on I/R-induced damage in the rodent retina. Thus, SF could be a potential preventative agent against oxidative stress, apoptotic activity, and inflammatory responses in retinal I/R injury. On the other hand, as demonstrated in our prior study, HO-1 expression levels were elevated more in SF- than LBP-treated I/R animals [24]. However, LBP-treated animals showed a significantly greater number of surviving RGCs (Figure 4E). This discrepancy could be a result of their opposite effects on the ERK pathway. Indeed, it was reported that LBP pretreatment suppressed the elevation of phosphorylated ERK1/2 [60], whereas SF induced ERK1/2 activation [61].

In conclusion, I/R induced a marked increase of ROS generation, caused pronounced inflammation, and increased the apoptosis of RGCs and amacrine cells. These effects were diminished or abolished by SF pretreatment. SF pretreatment significantly increased the nuclear accumulation of Nrf2 and HO-1 expression in I/R retinas. Together, these results suggest that neuroprotective effects of SF on I/R retinas were accomplished at least in part by the activation of the Nrf2/HO-1 antioxidant pathway.

## References

[1] Osborne NN, Casson RJ, Wood JPM, Chidlow G, Graham M, et al (2004) Retinal ischemia: mechanisms of damage and potential therapeutic strategies. Prog Retin Eye Res 23:91–147 Available: http://www.ncbi.nlm.nih.gov/pubmed/14766318. Accessed 22 September 2013.. 14766318 10.1016/j.preteyeres.2003.12.001

[2] Szabo ME, Haines D, Garay E, Chiavaroli C, Farine J-C, et al (2001) Antioxidant properties of calcium dobesilate in ischemic/reperfused diabetic rat retina. Eur J Pharmacol 428:277–286 Available: http://www.ncbi.nlm.nih.gov/pubmed/11675046. Accessed 22 September 2013.. 11675046 10.1016/s0014-2999(01)01196-7

[3] Kusari J, Padillo E, Zhou SX, Bai Y, Wang J, et al (2011) Effect of brimonidine on retinal and choroidal neovascularization in a mouse model of retinopathy of prematurity and laser-treated rats. Invest Ophthalmol Vis Sci 52:5424–5431 Available: http://www.pubmedcentral.nih.gov/articlerender.fcgi?artid=3176033&tool=pmcentrez&rendertype=abstract. Accessed 22 September 2013.. 21482645 10.1167/iovs.10-6262 PMC3176033

[4] Wei Y, Gong J, Yoshida T, Eberhart CG, Xu Z, et al (2011) Nrf2 has a protective role against neuronal and capillary degeneration in retinal ischemia-reperfusion injury. Free Radic Biol Med 51:216–224 Available: http://www.ncbi.nlm.nih.gov/pubmed/21545836. Accessed 13 August 2013.. 21545836 10.1016/j.freeradbiomed.2011.04.026 PMC3997112

[5] Hong SM, Yang YS (2012) A potential role of crystallin in the vitreous bodies of rats after ischemia-reperfusion injury. Korean J Ophthalmol 26:248–254 Available: http://www.pubmedcentral.nih.gov/articlerender.fcgi?artid=3408528&tool=pmcentrez&rendertype=abstract. Accessed 10 September 2013.. 22870022 10.3341/kjo.2012.26.4.248 PMC3408528

[6] Zhang Z, Qin X, Zhao X, Tong N, Gong Y, et al (2012) Valproic acid regulates antioxidant enzymes and prevents ischemia/reperfusion injury in the rat retina. Curr Eye Res 37:429–437 Available: http://www.ncbi.nlm.nih.gov/pubmed/22458760. 22458760 10.3109/02713683.2011.653616

[7] Owuor ED, Kong A-NT (2002) Antioxidants and oxidants regulated signal transduction pathways. Biochem Pharmacol 64:765–770 Available: http://www.sciencedirect.com/science/article/pii/S0006295202011371. Accessed 30 October 2013.. 12213568 10.1016/s0006-2952(02)01137-1

[8] Tsuji Y (2005) JunD activates transcription of the human ferritin H gene through an antioxidant response element during oxidative stress. Oncogene 24:7567–7578 Available: http://www.pubmedcentral.nih.gov/articlerender.fcgi?artid=2365508&tool=pmcentrez&rendertype=abstract. Accessed 30 October 2013.. 16007120 10.1038/sj.onc.1208901 PMC2365508

[9] Nerland DE (2007) The antioxidant/electrophile response element motif. Drug Metab Rev 39:235–248 Available: http://www.ncbi.nlm.nih.gov/pubmed/17364885. Accessed 30 October 2013.. 17364885 10.1080/03602530601125000

[10] Jeong W-S, Jun M, Kong A-NT (2006) Nrf2: a potential molecular target for cancer chemoprevention by natural compounds. Antioxid Redox Signal 8:99–106 Available: http://www.ncbi.nlm.nih.gov/pubmed/16487042. Accessed 24 September 2013.. 16487042 10.1089/ars.2006.8.99

[11] Pickering AM, Linder R a, Zhang H, Forman HJ, Davies KJ a (2012) Nrf2-dependent induction of proteasome and Pa28αβ regulator are required for adaptation to oxidative stress. J Biol Chem 287:10021–10031 Available: http://www.pubmedcentral.nih.gov/articlerender.fcgi?artid=3323025&tool=pmcentrez&rendertype=abstract. Accessed 22 September 2013.. 22308036 10.1074/jbc.M111.277145 PMC3323025

[12] McMahon M, Thomas N, Itoh K, Yamamoto M, Hayes JD (2004) Redox-regulated turnover of Nrf2 is determined by at least two separate protein domains, the redox-sensitive Neh2 degron and the redox-insensitive Neh6 degron. J Biol Chem 279:31556–31567 Available: http://www.ncbi.nlm.nih.gov/pubmed/15143058. Accessed 22 September 2013.. 15143058 10.1074/jbc.M403061200

[13] Itoh K, Wakabayashi N, Katoh Y (1999) Keap1 represses nuclear activation of antioxidant responsive elements by Nrf2 through binding to the amino-terminal Neh2 domain. Genes … 13:76–86 Available: http://genesdev.cshlp.org/content/13/1/76.short. Accessed 22 September 2013.. 10.1101/gad.13.1.76 PMC316370 9887101

[14] Satoh T, Okamoto S, Cui J, Watanabe Y, Furuta K, et al (2006) Activation of the Keap1/Nrf2 pathway for neuroprotection by electrophilic [correction of electrophillic] phase II inducers. Proc Natl Acad Sci U S A 103:768–773 Available: http://www.pubmedcentral.nih.gov/articlerender.fcgi?artid=1334635&tool=pmcentrez&rendertype=abstract. Accessed 11 September 2013.. 16407140 10.1073/pnas.0505723102 PMC1334635

[15] Kang KW, Lee SJ, Kim SG (2005) Molecular mechanism of nrf2 activation by oxidative stress. Antioxid Redox Signal 7:1664–1673 Available: http://www.ncbi.nlm.nih.gov/pubmed/16356128. Accessed 22 September 2013.. 16356128 10.1089/ars.2005.7.1664

[16] Kobayashi M, Yamamoto M (2005) Molecular mechanisms activating the Nrf2-Keap1 pathway of antioxidant gene regulation. Antioxid Redox Signal 7:385–394 Available: http://www.ncbi.nlm.nih.gov/pubmed/15706085. Accessed 22 September 2013.. 15706085 10.1089/ars.2005.7.385

[17] Fan J, Xu G, Jiang T, Qin Y (2012) Pharmacologic induction of heme oxygenase-1 plays a protective role in diabetic retinopathy in rats. Invest Ophthalmol Vis Sci 53:6541–6556 Available: http://www.ncbi.nlm.nih.gov/pubmed/22661484. Accessed 24 August 2013.. 22661484 10.1167/iovs.11-9241

[18] Ren J, Fan C, Chen N, Huang J, Yang Q (2011) Resveratrol pretreatment attenuates cerebral ischemic injury by upregulating expression of transcription factor Nrf2 and HO-1 in rats. Neurochem Res 36:2352–2362 Available: http://www.ncbi.nlm.nih.gov/pubmed/21850487. Accessed 10 September 2013.. 21850487 10.1007/s11064-011-0561-8

[19] Berberat PO, Katori M, Kaczmarek E, Anselmo D, Lassman C, et al (2003) Heavy chain ferritin acts as an antiapoptotic gene that protects livers from ischemia reperfusion injury. FASEB J 17:1724–1726 Available: http://www.ncbi.nlm.nih.gov/pubmed/12958189. Accessed 23 September 2013.. 12958189 10.1096/fj.03-0229fje

[20] Nakao A, Neto JS, Kanno S, Stolz DB, Kimizuka K, et al (2005) Protection against ischemia/reperfusion injury in cardiac and renal transplantation with carbon monoxide, biliverdin and both. Am J Transplant 5:282–291 Available: http://www.ncbi.nlm.nih.gov/pubmed/15643987. Accessed 23 September 2013.. 15643987 10.1111/j.1600-6143.2004.00695.x

[21] Poss KD, Tonegawa S (1997) Reduced stress defense in heme oxygenase 1-deficient cells. Proc Natl Acad Sci 94:10925–10930 Available: http://www.pubmedcentral.nih.gov/articlerender.fcgi?artid=23533&tool=pmcentrez&rendertype=abstract. Accessed 23 September 2013.. 9380736 10.1073/pnas.94.20.10925 PMC23533

[22] Sun M-H, Pang J-HS, Chen S-L, Han W-H, Ho T-C, et al (2010) Retinal protection from acute glaucoma-induced ischemia-reperfusion injury through pharmacologic induction of heme oxygenase-1. Invest Ophthalmol Vis Sci 51:4798–4808 Available: http://www.ncbi.nlm.nih.gov/pubmed/20357190. Accessed 10 September 2013.. 20357190 10.1167/iovs.09-4086

[23] Arai-Gaun S, Katai N, Kikuchi T, Kurokawa T, Ohta K, et al (2004) Heme oxygenase-1 induced in muller cells plays a protective role in retinal ischemia-reperfusion injury in rats. Invest Ophthalmol Vis Sci 45:4226–4232 Available: http://www.ncbi.nlm.nih.gov/pubmed/15505079 Accessed 23 September 2013.. 15505079 10.1167/iovs.04-0450

[24] He M, Pan H, Chang RC-C, So K-F, Brecha NC, et al (2014) Activation of the Nrf2/HO-1 Antioxidant Pathway Contributes to the Protective Effects of Lycium Barbarum Polysaccharides in the Rodent Retina after Ischemia- Reperfusion-Induced Damage. PLoS One 9:e84800 doi:10.1371/journal.pone.0084800. eCollection 2014 24400114 10.1371/journal.pone.0084800 PMC3882254

[25] Zhang Y, Talalay P, Cho CG, Posner GH (1992) A major inducer of anticarcinogenic protective enzymes from broccoli: isolation and elucidation of structure. Proc Natl Acad Sci U S A 89:2399–2403 Available: http://www.pubmedcentral.nih.gov/articlerender.fcgi?artid=48665&tool=pmcentrez&rendertype=abstract. 1549603 10.1073/pnas.89.6.2399 PMC48665

[26] Piao CS, Gao S, Lee G-H, Kim DS, Park B-H, et al (2010) Sulforaphane protects ischemic injury of hearts through antioxidant pathway and mitochondrial K(ATP) channels. Pharmacol Res 61:342–348 Available: http://www.ncbi.nlm.nih.gov/pubmed/19948220 Accessed 29 August 2013.. 19948220 10.1016/j.phrs.2009.11.009

[27] Zhao H-D (2010) Sulforaphane protects liver injury induced by intestinal ischemia reperfusion through Nrf2-ARE pathway. World J Gastroenterol 16:3002 Available: http://www.ncbi.nlm.nih.gov/pubmed/23932610 Accessed 11 September 2013.. 20572303 10.3748/wjg.v16.i24.3002 PMC2890940

[28] Ping Z, Liu W, Kang Z, Cai J, Wang Q, et al (2010) Sulforaphane protects brains against hypoxic-ischemic injury through induction of Nrf2-dependent phase 2 enzyme. Brain Res 1343:178–185 Available: http://www.ncbi.nlm.nih.gov/pubmed/20417626 Accessed 7 November 2013.. 20417626 10.1016/j.brainres.2010.04.036

[29] Yoon H-Y, Kang N-I, Lee H-K, Jang KY, Park J-W, et al (2008) Sulforaphane protects kidneys against ischemia-reperfusion injury through induction of the Nrf2-dependent phase 2 enzyme. Biochem Pharmacol 75:2214–2223 Available: http://www.ncbi.nlm.nih.gov/pubmed/20417626 Accessed 12 September 2013.. 18407246 10.1016/j.bcp.2008.02.029

[30] Gao X, Talalay P (2004) Induction of phase 2 genes by sulforaphane protects retinal pigment epithelial cells against photooxidative damage. Proc Natl Acad Sci U S A 101:10446–10451 Available: http://www.pubmedcentral.nih.gov/articlerender.fcgi?artid=478590&tool=pmcentrez&rendertype=abstract Accessed 11 September 2013.. 15229324 10.1073/pnas.0403886101 PMC478590

[31] Tanito M, Masutani H, Kim Y-C, Nishikawa M, Ohira A, et al (2005) Sulforaphane induces thioredoxin through the antioxidant-responsive element and attenuates retinal light damage in mice. Invest Ophthalmol Vis Sci 46:979–987 Available: http://www.ncbi.nlm.nih.gov/pubmed/15728556 Accessed 19 October 2013.. 15728556 10.1167/iovs.04-1120

[32] Zheng H, Whitman SA, Wu W, Wondrak GT, Wong PK, et al (2011) Therapeutic potential of Nrf2 activators in streptozotocin-induced diabetic nephropathy. Diabetes 60:3055–3066 Available: http://www.pubmedcentral.nih.gov/articlerender.fcgi?artid=3198067&tool=pmcentrez&rendertype=abstract Accessed 24 August 2013.. 22025779 10.2337/db11-0807 PMC3198067

[33] Park J, Kang J-W, Lee S-M (2013) Activation of the cholinergic anti-inflammatory pathway by nicotine attenuates hepatic ischemia/reperfusion injury via heme oxygenase-1 induction. Eur J Pharmacol 707:61–70 Available: http://www.sciencedirect.com/science/article/pii/S0014299913002161 Accessed 23 September 2013.. 23535606 10.1016/j.ejphar.2013.03.026

[34] He M, Pan H, Xiao C, Pu M (2013) Roles for redox signaling by NADPH oxidase in hyperglycemia-induced heme oxygenase-1 expression in the diabetic retina. Invest Ophthalmol Vis Sci 54:4092–4101 Available: http://www.ncbi.nlm.nih.gov/pubmed/23633655 Accessed 26 September 2013.. 23633655 10.1167/iovs.13-12004

[35] Gunhan E, Choudary P V, Landerholm TE, Chalupa LM (2002) Depletion of cholinergic amacrine cells by a novel immunotoxin does not perturb the formation of segregated on and off cone bipolar cell projections. J Neurosci 22:2265–2273 Available: http://www.ncbi.nlm.nih.gov/pubmed/11896166. 11896166 10.1523/JNEUROSCI.22-06-02265.2002 PMC6758270

[36] Kwong JMK, Quan A, Kyung H, Piri N, Caprioli J (2011) Quantitative analysis of retinal ganglion cell survival with Rbpms immunolabeling in animal models of optic neuropathies. Invest Ophthalmol Vis Sci 52:9694–9702 Available: http://www.pubmedcentral.nih.gov/articlerender.fcgi?artid=3341125&tool=pmcentrez&rendertype=abstract Accessed 23 September 2013.. 22110060 10.1167/iovs.11-7869 PMC3341125

[37] Hörnberg H, Wollerton-van Horck F, Maurus D, Zwart M, Svoboda H, et al (2013) RNA-binding protein Hermes/RBPMS inversely affects synapse density and axon arbor formation in retinal ganglion cells in vivo. J Neurosci 33:10384–10395 Available: http://www.ncbi.nlm.nih.gov/pubmed/23785151 Accessed 23 September 2013.. 23785151 10.1523/JNEUROSCI.5858-12.2013 PMC4603358

[38] Mi X-S, Feng Q, Lo ACY, Chang RC-C, Lin B, et al (2012) Protection of retinal ganglion cells and retinal vasculature by Lycium barbarum polysaccharides in a mouse model of acute ocular hypertension. PLoS One 7:e45469 Available: http://www.pubmedcentral.nih.gov/articlerender.fcgi?artid=3477168&tool=pmcentrez&rendertype=abstract Accessed 24 August 2013.. 23094016 10.1371/journal.pone.0045469 PMC3477168

[39] Sakamoto K, Kawakami T, Shimada M, Yamaguchi A, Kuwagata M, et al (2009) Histological protection by cilnidipine, a dual L/N-type Ca(2+) channel blocker, against neurotoxicity induced by ischemia-reperfusion in rat retina. Exp Eye Res 88:974–982 Available: http://www.ncbi.nlm.nih.gov/pubmed/19166832 Accessed 25 September 2013.. 19166832 10.1016/j.exer.2008.12.011

[40] Da T, Verkman a S (2004) Aquaporin-4 gene disruption in mice protects against impaired retinal function and cell death after ischemia. Invest Ophthalmol Vis Sci 45:4477–4483 Available: http://www.ncbi.nlm.nih.gov/pubmed/15557457 Accessed 25 September 2013.. 15557457 10.1167/iovs.04-0940

[41] Wang L, Li C, Guo H, Kern TS, Huang K, et al (2011) Curcumin inhibits neuronal and vascular degeneration in retina after ischemia and reperfusion injury. PLoS One 6:e23194 Available: http://www.pubmedcentral.nih.gov/articlerender.fcgi?artid=3153496&tool=pmcentrez&rendertype=abstract Accessed 24 August 2013.. 21858029 10.1371/journal.pone.0023194 PMC3153496

[42] Liu Y, Tang L, Chen B (2012) Effects of antioxidant gene therapy on retinal neurons and oxidative stress in a model of retinal ischemia/reperfusion. Free Radic Biol Med 52:909–915 Available: http://www.ncbi.nlm.nih.gov/pubmed/22240151 Accessed 15 October 2013.. 22240151 10.1016/j.freeradbiomed.2011.12.013

[43] Fang I-M, Yang C-M, Yang C-H, Chiou S-H, Chen M-S (2013) Transplantation of induced pluripotent stem cells without C-Myc attenuates retinal ischemia and reperfusion injury in rats. Exp Eye Res 113:49–59 Available: http://linkinghub.elsevier.com/retrieve/pii/S0014483513001267 Accessed 9 September 2013.. 23726881 10.1016/j.exer.2013.05.007

[44] Oharazawa H, Igarashi T, Yokota T, Fujii H, Suzuki H, et al (2010) Protection of the retina by rapid diffusion of hydrogen: administration of hydrogen-loaded eye drops in retinal ischemia-reperfusion injury. Invest Ophthalmol Vis Sci 51:487–492 Available: http://www.ncbi.nlm.nih.gov/pubmed/19834032 Accessed 15 October 2013.. 19834032 10.1167/iovs.09-4089

[45] Shima C, Adachi Y, Minamino K, Okigaki M, Shi M, et al (2012) Neuroprotective effects of granulocyte colony-stimulating factor on ischemia-reperfusion injury of the retina. Ophthalmic Res 48:199–207 Available: http://www.ncbi.nlm.nih.gov/pubmed/23728838 Accessed 7 November 2013.. 22868688 10.1159/000340059

[46] Li J-B, Lu Z-G, Xu L, Wang Q, Zhang Z-H, et al (2013) Neuroprotective Effects of Bis(7)-tacrine in a Rat Model of Pressure-Induced Retinal Ischemia. Cell Biochem Biophys 68:275–282 Available: http://www.ncbi.nlm.nih.gov/pubmed/23832279 Accessed 7 November 2013.. 10.1007/s12013-013-9707-4 23832279

[47] Dvoriantchikova G, Grant J, Santos ARC, Hernandez E, Ivanov D (2012) Neuronal NAD(P)H oxidases contribute to ROS production and mediate RGC death after ischemia. Invest Ophthalmol Vis Sci 53:2823–2830 Available: http://www.ncbi.nlm.nih.gov/pubmed/22467573 Accessed 10 September 2013.. 22467573 10.1167/iovs.12-9526 PMC4686185

[48] Ishizuka F, Shimazawa M, Inoue Y, Nakano Y, Ogishima H, et al (2013) Toll-Like Receptor 4 Mediates Retinal Ischemia/Reperfusion Injury Through Nuclear Factor-κB and Spleen Tyrosine Kinase Activation. Invest Ophthalmol Vis Sci 54:5807–5816 Available: http://www.iovs.org/content/54/8/5807.short Accessed 15 October 2013.. 23908181 10.1167/iovs.13-11932

[49] Alp H, Aytekin I, Hatipoglu NK, Alp A, Ogun M (2012) Effects of sulforophane and curcumin on oxidative stress created by acute malathion toxicity in rats. Eur Rev Med Pharmacol Sci 16 Suppl 3: 144–148 Available: http://www.europeanreview.org/wp/wp-content/uploads/1156.pdf Accessed 7 November 2013.. 22957429

[50] Zhang Y, Kensler TW, Cho CG, Posner GH, Talalay P (1994) Anticarcinogenic activities of sulforaphane and structurally related synthetic norbornyl isothiocyanates. Proc Natl Acad Sci U S A 91:3147–3150 Available: http://www.ncbi.nlm.nih.gov/pubmed/18591798. 8159717 10.1073/pnas.91.8.3147 PMC43532

[51] Cekauskas A, Bruns H, Manikas M, Herr I, Gross M-L, et al (2013) Sulforaphane decreases kidney injury after transplantation in rats: role of mitochondrial damage. Ann Transplant 18:488–496 Available: http://www.ncbi.nlm.nih.gov/pubmed/24048440 Accessed 24 September 2013.. 24048440 10.12659/AOT.884013

[52] Guerrero-Beltrán CE, Mukhopadhyay P, Horváth B, Rajesh M, Tapia E, et al (2012) Sulforaphane, a natural constituent of broccoli, prevents cell death and inflammation in nephropathy. J Nutr Biochem 23:494–500 Available: http://www.pubmedcentral.nih.gov/articlerender.fcgi?artid=3179776&tool=pmcentrez&rendertype=abstract Accessed 16 October 2013.. 21684138 10.1016/j.jnutbio.2011.02.004 PMC3179776

[53] Yang L-P, Zhu X-A, Tso MOM (2007) Role of NF-kappaB and MAPKs in light-induced photoreceptor apoptosis. Invest Ophthalmol Vis Sci 48:4766–4776 Available: http://www.ncbi.nlm.nih.gov/pubmed/17898303 Accessed 19 October 2013.. 17898303 10.1167/iovs.06-0871

[54] Kleszczyński K, Ernst IMA, Wagner AE, Kruse N, Zillikens D, et al (2013) Sulforaphane and phenylethyl isothiocyanate protect human skin against UVR-induced oxidative stress and apoptosis: Role of Nrf2-dependent gene expression and antioxidant enzymes. Pharmacol Res 78C:28–40 Available: http://www.ncbi.nlm.nih.gov/pubmed/24121007 Accessed 7 November 2013.. 10.1016/j.phrs.2013.09.009 24121007

[55] Kong L, Tanito M, Huang Z, Li F, Zhou X, et al (2007) Delay of photoreceptor degeneration in tubby mouse by sulforaphane. J Neurochem 101:1041–1052 Available: http://www.ncbi.nlm.nih.gov/pubmed/17394579 Accessed 19 October 2013.. 17394579 10.1111/j.1471-4159.2007.04481.x

[56] Chao H-M, Chuang M-J, Liu J-H, Liu X-Q, Ho L-K, et al (2013) Baicalein Protects Against Retinal Ischemia by Antioxidation, Antiapoptosis, Downregulation of HIF-1α, VEGF, and MMP-9 and Upregulation of HO-1. J Ocul Pharmacol Ther 29:539–549 Available: http://online.liebertpub.com/doi/abs/10.1089/jop.2012.0179 Accessed 9 September 2013.. 23537149 10.1089/jop.2012.0179 PMC3708628

[57] Kaur C, Foulds WS, Ling EA (2008) Blood-retinal barrier in hypoxic ischaemic conditions: basic concepts, clinical features and management. Prog Retin Eye Res 27:622–647 Available: http://www.ncbi.nlm.nih.gov/pubmed/18940262 Accessed 24 September 2013.. 18940262 10.1016/j.preteyeres.2008.09.003

[58] Leal EC, Martins J, Voabil P, Liberal J, Chiavaroli C, et al (2010) Calcium dobesilate inhibits the alterations in tight junction proteins and leukocyte adhesion to retinal endothelial cells induced by diabetes. Diabetes 59:2637–2645 Available: http://www.pubmedcentral.nih.gov/articlerender.fcgi?artid=3279541&tool=pmcentrez&rendertype=abstract Accessed 24 September 2013.. 20627932 10.2337/db09-1421 PMC3279541

[59] Shah Z a, Li R, Ahmad AS, Kensler TW, Yamamoto M, et al (2010) The flavanol (−)-epicatechin prevents stroke damage through the Nrf2/HO1 pathway. J Cereb Blood Flow Metab 30:1951–1961 Available: http://www.pubmedcentral.nih.gov/articlerender.fcgi?artid=3002885&tool=pmcentrez&rendertype=abstract Accessed 24 September 2013.. 20442725 10.1038/jcbfm.2010.53 PMC3002885

[60] Ho Y-S, Yu M-S, Yang X-F, So K-F, Yuen W-H, et al (2010) Neuroprotective effects of polysaccharides from wolfberry, the fruits of Lycium barbarum, against homocysteine-induced toxicity in rat cortical neurons. J Alzheimers Dis 19:813–827 Available: http://www.ncbi.nlm.nih.gov/pubmed/20157238 Accessed 14 March 2014.. 20157238 10.3233/JAD-2010-1280

[61] Guerrero-Beltrán CE, Mukhopadhyay P, Horváth B, Rajesh M, Tapia E, et al (2012) Sulforaphane, a natural constituent of broccoli, prevents cell death and inflammation in nephropathy. J Nutr Biochem 23:494–500 Available: http://www.pubmedcentral.nih.gov/articlerender.fcgi?artid=3179776&tool=pmcentrez&rendertype=abstract Accessed 12 March 2014.. 21684138 10.1016/j.jnutbio.2011.02.004 PMC3179776

